# Association of the Polymorphisms in the *Fas/FasL* Promoter Regions with Cancer Susceptibility: A Systematic Review and Meta-Analysis of 52 Studies

**DOI:** 10.1371/journal.pone.0090090

**Published:** 2014-03-05

**Authors:** Yeqiong Xu, Bangshun He, Rui Li, Yuqin Pan, Tianyi Gao, Qiwen Deng, Huiling Sun, Guoqi Song, Shukui Wang

**Affiliations:** 1 Central Laboratory, Nanjing First Hospital, Nanjing Medical University, Nanjing, China; 2 Department of Life Sciences, Nanjing Normal University, Nanjing, China; Duke Cancer Institute, United States of America

## Abstract

Fas and its ligand (FasL) play an important role in apoptosis and carcinogenesis. Therefore, the potential association of polymorphisms in the *Fas* (-670A>G, rs1800682; -1377G>A, rs2234767) and *FasL* (-844C>T, rs763110) with cancer risk has been widely investigated. However, all the currently available results are not always consistent. In this work, we performed a meta-analysis to further determine whether carriers of the polymorphisms in *Fas* and *FasL* of interest could confer an altered susceptibility to cancer. All relevant data were retrieved by PubMed and Web of Science, and 52 eligible studies were chosen for this meta-analysis. There was no association of the *Fas* -670A>G polymorphism with cancer risk in the pooled data. For the *Fas* -1377G>A and *FasL* -844C>T polymorphisms, results revealed that the homozygotes of -1377A and -844C were associated with elevated risk of cancer as a whole. Further stratified analysis indicated markedly increased risk for developing breast cancer, gastric cancer, and esophageal cancer, in particular in Asian population. We conclude that carriers of the *Fas*-1377A and the *FasL* -844C are more susceptible to the majority of cancers than non-carriers.

## Introduction

With new cases and mortality increased dramatically, cancer has become the major public health burden worldwide. For this reason, novel diagnostic markers are needed urgently for early detection and prevention of cancer. However, carcinogenesis is a complicated biological process that is not fully understood. It is generally believed that interactions of low-penetrance susceptibility genes with environmental factors might contribute to carcinogenesis [1]. As one of the important low-penetrance genes, *Fas* is considered to be a potential cancer susceptibility gene. This is because Fas (TNFSF6, CD95, or APO-1) is a cell surface receptor involved in apoptotic signal transmission in many cell types and interacts with its natural ligand Fas ligand (also known as FasL) to initiate the death signal cascade that leads to apoptotic cell death [2], [3]. Furthermore, in these two genes, there are several functionally significant polymorphisms, such as the −670A>G and −1377G>A in the *Fas* promoter region, and the −844C>T in the *FasL* promoter region, because they might be associated with cancer risk, including cervical cancer [4]–[9], gastric cancer [10]–[15], breast cancer [16]–[21], lung cancer [22]–[25] and so on. However, all available results are not always consistent with one another, partially because of the small sample size of some published studies, different ethnic backgrounds, publication bias, and little effect of the polymorphisms on cancer risk. Therefore, it's necessary to retrieve and pool all eligible data to further determine whether these genetic polymorphisms could be at increased risk for developing cancer and to what extent heterogeneity existed across all the studies.

## Materials and Methods

### Identification and eligibility of relevant studies

Two online medical databases, PubMed, and Web of Science, were searched (updated February 2013), using the search terms “Fas/CD95/TNFSF6/APO-1”, “FasL/CD95L”, “polymorphism/genetic variation” and “cancer/carcinoma/tumor”). The literature search was limited to English articles. In addition, more studies were also identified by manual search based on the references provided in the retrieved studies. The inclusion criteria were prespecified as below: (1) be a case-control study, (2) evaluate association between the *Fas* and/or *FasL* polymorphisms and cancer risk, (3) present sufficient data to calculate an odds ratio (OR) with 95% confidence interval (CI), and (4) list genotype frequency. Moreover, the studies without raw data, or those that were case-only studies, case reports, editorials, and review articles (including meta-analyses) were eliminated.

### Data extraction

Information was extracted carefully from all eligible articles independently by two authors (Yeqiong Xu and Bangshun He) according to the above inclusion and exclusion criteria. Discrepancies were resolved by extensive discussion in our research team. The characteristics of enrolled studies were extracted as below: the first author's last name, year of publication, country of subjects, ethnicity, type of cancer, the source of controls, genotyping method (whether PCR was performed using a dual-labelled TaqMan probe with a specific 3'base to detect the SNPs or whether an RFLP method was used), the number of matched cases and controls, polymorphism sites, and *P* value for Hardy–Weinberg equilibrium (HWE) as summarized in Table 1.

**Table 1:** Characteristics of studies included in the meta-analysis.

Cancer type	Year	First author	Country	Ethnicity	Source of control	Genotyping method	Polymorphism sites	Cases	Controls	HWE
Cervical cancer										
	2009	Zucchini [52]	Maton Grosso do Sul, Brazil	African	PB	PCR-RFLP	*Fas* -670A>G	91	176	0.545
	2008	Tamandani [35]	Northern India	Asian	HB	PCR-RFLP	*Fas* -670A>G	200	200	0.001
	2008	Kang [4]	Korea	Asian	PB	PCR-RFLP	*Fas* -670A>G, *Fas* -1377G>A, *FasL* -844C>T	154	160	0.264, 0.233, 0.327
	2007	Ivansson [53]	Sweden	Caucasian	PB	TaqMan	*FasL* -844C>T	1284	280	0.738
	2006	Ueda [34]	Japan	Asian	PB	PCR-RFLP	*Fas* -670A>G	83	95	0.172
	2005	Zoodsma [5]	Netherlands	Caucasian	PB	TaqMan	*Fas* -670A>G	670	607	0.274
	2005	Sun [6]	China	Asian	PB	PCR-RFLP	*Fas* -670A>G, *Fas* -1377G>A, *FasL* -844C>T	314	615	0.641, 0.304, 0.002
	2005	Lai [7]	China	Asian	HB	TaqMan	*Fas* -670A>G, *Fas* -1377G>A, *FasL* -844C>T	318	318	0.736, 0.293, 0.920
	2004	Dybikowska [8]	Poland	Caucasian	PB	PCR-RFLP	*Fas* -670A>G	51	65	0.638
	2003	Lai [9]	China	Asian	HB	PCR-RFLP	*Fas* -670A>G	176	176	0.444
Gastric cancer										
	2012	Zhang [10]	China	Asian	HB	PCR-RFLP	*Fas* -1377G>A, *FasL* -844C>T	375	496	0.064, 0.112
	2011	Liu [12]	China	Asian	PB	PCR-RFLP	*Fas* -1377G>A, *FasL* -844C>T	344	324	0.424, 0.083
	2011	Kupcinskas [11]	Mixed	Caucasian	PB	TaqMan	*Fas* -670A>G, *Fas* -1377G>A, *FasL* -844C>T	114	238	0.199, 0.492, 0.715
	2010	Zhou [13]	China	Asian	PB	PCR-RFLP	*Fas* -670A>G, *Fas* -1377G>A, *FasL* -844C>T	262	524	0.133, 0.062, 0.899
	2009	Wang [14]	China	Asian	PB	PCR-RFLP	*Fas* -670A>G, *Fas* -1377G>A, *FasL* -844C>T	332	324	0.806, 0.870, 0.554
	2008	Hsu [15]	China	Asian	PB	PCR-RFLP	*Fas* -670A>G, *Fas* -1377G>A, *FasL* -844C>T	86	101	0.736, 0.914, 0.612
	2006	Ikehara [54]	Japan	Asian	PB	PCR-CTPP	*Fas* -670A>G	271	271	0.504
Breast cancer										
	2013	Hashemi [16]	Iranian	Caucasian	PB	T-ARMS-PCR	*Fas* -670A>G, *Fas* -1377G>A, *FasL* -844C>T	134	152	0.045, 0.000, 0.183
	2012	Wang [17]	China	Asian	HB	PCR-RFLP	*Fas* -1377G>A, *FasL* -844C>T	375	496	0.064, 0.112
	2012	Mahfoudh [18]	Tunisia	African	PB	PCR-RFLP	*FasL* -844C>T	438	332	0.334
	2007	Crew [19]	America	Caucasian	PB	TaqMan	*Fas* -670A>G, *Fas* -1377G>A, *FasL* -844C>T	1051	1101	0.754, 0.069, 0.602
	2007	Zhang [20]	China	Asian	HB	PCR-RFLP	*Fas* -670A>G, *Fas* -1377G>A, *FasL* -844C>T	836	834	0.797, 0.700, 0.110
	2004	Krippl [21]	Austria	Caucasian	PB	TaqMan	*Fas* -670A>G, *Fas* -1377G>A, *FasL* -844C>T	499	495	0.924, 0.610, 0.418
Lung cancer										
	2008	Ter-Minassian [22]	America	Caucasian	HB	TaqMan	*Fas* -1377G>A, *FasL* -844C>T	2174	1497	0.751, 0.254
	2007	Gormus [23]	Turkey	Caucasian	PB	PCR-RFLP	*Fas* -1377G>A	94	50	0.000
	2006	Park [24]	Korea	Asian	PB	PCR-RFLP	*Fas* -670A>G, *Fas* -1377G>A, *FasL* -844C>T	582	582	0.132, 0.024, 0.570
	2005	Zhang [25]	China	Asian	PB	PCR-RFLP	*Fas* -1377G>A, *FasL* -844C>T	1000	1270	0.046, 0.180
	2003	Wang [55]	America	Mixed	PB	PCR-RFLP	*Fas* -670A>G	68	74	0.481
Esophageal cancer										
	2011	Bye [32]	Eastern or Western Cape	African	PB	TaqMan	*Fas* -670A>G, *Fas* -1377G>A, *FasL* -844C>T	343	466	0.027, 0.670, 0.097
	2011	Bye [32]	Western Cape	Mixed	PB	TaqMan	*Fas* -670A>G, *Fas* -1377G>A, *FasL* -844C>T	195	420	0.170, 0.469, 0.741
	2007	Jain [56]	Northern India	Asian	PB	PCR-RFLP	*Fas -670A>G*	151	201	0.140
	2003	Sun [57]	China	Asian	PB	PCR-RFLP	*Fas* -670A>G, *Fas* -1377G>A, *FasL* -844C>T	588	648	0.130, 0.218, 0.061
Skin cancer										
	2010	Qureshi [58]	Britain	Caucasian	PB	NA	*Fas* -670A>G, *Fas* -1377G>A, *FasL* -844C>T	779	842	0.210, 0.916, 0.427
	2007	Zhang [59]	Sweden	Caucasian	PB	PCR-RFLP	*Fas* -670A>G, *Fas* -1377G>A, *FasL* -844C>T	229	351	0.380, 0.009, 0.609
	2006	Li [60]	America	Caucasian	HB	PCR-RFLP	*Fas* -670A>G, *Fas* -1377G>A, *FasL* -844C>T	602	603	0.453, 0.951, 0.071
	2001	Nelson [61]	America	Caucasian	PB	PCR-RFLP	*Fas* -670A>G	776	435	0.117
Ovarian cancer										
	2012	Li [62]	China	Asian	PB	Allele-specific multiple ligase detection	*Fas* -670A>G, *Fas* -1377G>A, *FasL* -844C>T	342	344	0.357, 0.972, 0.547
	2007	Gormus [63]	Turkey	Caucasian	PB	PCR-RFLP	*Fas* -1377G>A, *FasL* -844C>T	47	41	0.272, 0.678
	2006	Ueda [34]	Japan	Asian	PB	PCR-RFLP	*Fas* -670A>G	68	95	0.172
Prostate cancer										
	2012	Mandal [51]	Northern India	Asian	HB	PCR-RFLP	*Fas* -670A>G, *Fas* -1377G>A	192	224	0.296, 0.035
	2011	Shao [38]	China	Asian	HB	PCR-RFLP	*Fas* -670A>G, *Fas* -1377G>A, *FasL* -844C>T	602	703	0.579, 0.099, 0.801
	2008	Lima [64]	Portugal	Caucasian	PB	PCR-RFLP	*Fas* -670A>G	657	247	0.365
Nasopharyngeal cancer										
	2010	Cao [65]	China	Asian	PB	PCR-RFLP	*Fas* -1377G>A, *FasL* -844C>T	576	608	0.984, 0.015
	2010	Zhu [66]	China	Asian	PB	PCR-RFLP	*Fas* -670A>G	237	264	0.478
	2006	Jrad [37]	Tunisia	African	PB	PCR-RFLP	*Fas* -670A>G	170	224	0.585
Bladder cancer										
	2010	Gangwar [67]	Northern India	Asian	PB	PCR-RFLP	*Fas* -670A>G	212	250	0.384
	2006	Li [68]	China	Asian	HB	PCR-RFLP	*Fas* -670A>G, *Fas* -1377G>A, *FasL* -844C>T	216	252	0.409, 0.970, 0.234
Other cancers										
	2010	Zhu [69]	China	Asian	HB	PCR-RFLP	*Fas* -670A>G, *Fas* -1377G>A, *FasL* -844C>T	353	365	0.831, 0.777, 0.278
	2010	Wang [70]	China	Asian	PB	PCR-RFLP	*Fas* -670A>G, *Fas* -1377G>A, *FasL* -844C>T	294	333	0.034, 0.628, 0.271
	2008	Yang [39]	China	Asian	PB	PCR-RFLP	*Fas* -670A>G, *Fas* -1377G>A, *FasL* -844C>T	397	907	0.653, 0.062, 0.986
	2007	Koshkina [71]	America	Mixed	PB	PCR-RFLP	*Fas* -670A>G, *Fas* -1377G>A	123	510	0.786, 0.210
	2007	Erdogan [72]	Turkey	Caucasian	HB	PCR-RFLP	*Fas* -670A>G, *FasL* -844C>T	45	100	0.812, 0.727
	2007	Ho^a^ [33]	America	Mixed	HB	PCR-RFLP	*Fas* -1377G>A	279	510	0.210
	2007	Ho^b^ [33]	America	Mixed	HB	PCR-RFLP	*Fas* -1377G>A	154	510	0.210
	2006	Zhang [36]	America	Caucasian	HB	PCR-RFLP	*Fas* -670A>G, *Fas* -1377G>A, *FasL* -844C>T	721	1234	0.481, 0.268, 0.411
	2006	Ueda [34]	Japan	Asian	PB	PCR-RFLP	*Fas* -670A>G	108	95	0.172

The Ho(^a^) investigated thyroid cancer, and the Ho(^b^) investigated salivary gland cancer.

PB: population based; HB: hospital based; T-ARMS-PCR:tetra-primeramplification refractory mutation system PCR; PCR-RFLP: restriction fragment length polymorphism; HWE: Hardy-Weinberg equilibrium.

### Genotype-gene expression correlation analysis

The International HapMap Project (http://hapmap.ncbi.nlm.nih.gov/) was used to obtain data of the *Fas* and *FasL* genotypes determined in 270 enrolled subjects. Meanwhile, the mRNA expression data of these enrolled subjects were available online from SNPexp (http://app3.titan.uio.no/biotools/help.php?app=snpexp) as described in the previous studies [26], [27]. In brief, these data were obtained from the HapMap phase II release 23 data set consisting of 3.96 million SNP genotypes from 270 subjects of three populations, including 90 European (CEU), 90 Asian (45 Chinese, 45 Japanese), and 90 Yoruba (YRI) subjects [28]. Additionally, the mRNA expression data were derived from the lymphoblastic cell lines from the same 270 subjects [29].

### Statistical analysis

Crude ORs with 95% CIs were used to assess the strength of association between the polymorphisms in *Fas*-670A>G, *Fas* -1377G>A, and *FasL* -844T/C and cancer risk. The pooled ORs were estimated for dominant model (variant homozygotes + heterozygous vs homozygous reference), recessive model (variant homozygotes vs heterozygous + homozygous reference), homozygote comparison (variant homozygotes vs homozygous reference), heterozygote comparison (heterozygous vs homozygous reference) and allelic comparison in the polymorphisms, respectively. Stratified analyses were performed by the type of cancer (that with only one study was grouped together as ‘other cancers’), ethnicity, source of controls and genotyping method. Heterogeneity across the studies was evaluated by using the Chi-square test based Q-statistic test, and it was considered statistically significant when *P_heterogeneity_* (*P_h_*)<0.05. The data were combined using random-effects model (the DerSimonian and Laird method) [30] in the presence of heterogeneity (*P*<0.05 or *I^2^*>50%), or fixed-effects model (the Mantel-Haenszel method) models [31] was chosen to use in the absence of heterogeneity (*P*>0.05 or *I^2^*<50%). Moreover, sensitivity analyses were performed to assess the stability of the results. Publication bias was evaluated graphically by using funnel plots and statistically by the Egger's linear regression test. HWE of the three polymorphisms was assessed using a web-based program (http://ihg.gsf.de/cgi-bin/hw/hwa1.pl). All statistical tests were performed with STATA 11.0 and SPSS 20.0. All the *P* values were two-sided.

## Results

**Figure 1:**
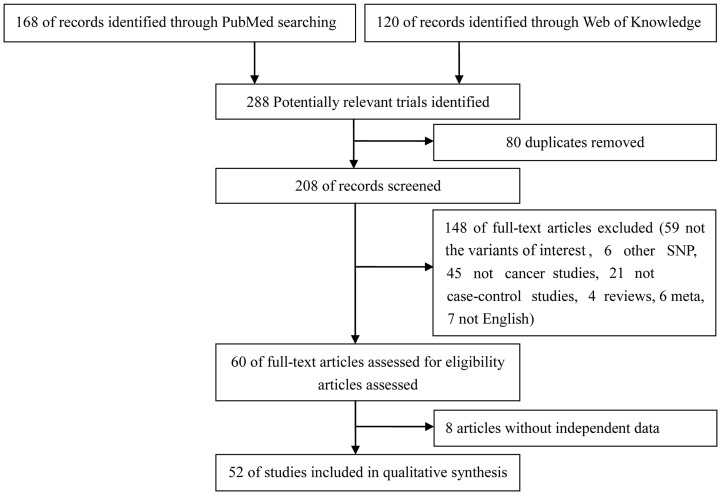
Flow chart of studies identified according to inclusion and exclusion criteria.

A total of 52 studies were enrolled in this meta-analysis (Figure 1). The major characteristics of the 52 selected studies are summarized in Table 1. The study carried out by Bye et al [32] analyzed individuals of African or Mixed ethnicity, and thus was divided into two studies. Similarly, the studies reported by Ho et al [33] and Ueda et al [34] investigated two and three types of cancer, and therefore, these two studies were cited as two studies and three studies, respectively (Table 1).

For the *Fas* -670A>G polymorphism, there was no association in the pooled analysis. In the subgroup analysis, statistically significantly decreased risk was observed in prostate cancer and melanoma for GG+AG vs AA comparison model, whereas there was significantly increased risk among those of African ancestry for GG+AG vs AA models (all data shown in Table 2).

**Table 2:** Stratified analyses of the *Fas* -670A>G (rs1800682) polymorphism and cancer.

Variables	n^a^	GG+AG vs AA			GG vs AG+AA			G vs A		
		OR(95%CI)	*P* b	*I^2^*	OR(95%CI)	*P* b	*I^2^*	OR(95%CI)	*P* b	*I^2^*
Total	44	1.01(0.94, 1.09)^c^	<0.0001	47.1	1.04(0.96, 1.12)^c^	0.003	40.9	1.02(0.97,1.06)^c^	0.005	39.4
Cancer type										
Cervical cancer	9	1.05(0.79,1.40)^c^	<0.0001	74.5	0.92(0.69, 1.22)^c^	0.006	62.8	0.99(0.86,1.14)^c^	0.013	58.5
Gastric cancer	5	1.08(0.91,1.28)	0.340	11.6	0.97(0.79,1.21)	0.978	0.0	1.03(0.91,1.15)	0.735	0.0
Esophageal cancer	4	1.02(0.85,1.21)	0.459	0.0	1.21(0.86,1.69)^c^	0.017	70.4	1.10(0.99,1.23)	0.215	32.9
Breast cancer	4	1.01(0.90,1.14)	0.325	13.4	1.03(0.90,1.18)	0.062	59.1	1.02(0.94,1.10)	0.259	25.5
Prostate cancer	3	**0.83(0.70,0.98)**	0.155	46.4	0.82(0.66,1.01)	0.346	5.8	0.87(0.77,0.97)	0.163	44.8
Ovarian cancer	2	0.87(0.66,1.15)	0.952	0.0	0.85(0.57,1.28)	0.622	0.0	0.90(0.74,1.09)	0.745	0.0
Bladder cancer	2	1.01(0.77,1.33)	0.588	0.0	1.00(0.47,2.16)^c^	0.043	75.6	1.03(0.85,1.24)	0.491	0.0
Skin cancer	2	1.08(0.91,1.27)	0.414	0.0	1.02(0.86,1.23)	0.483	0.0	1.04(0.93,1.16)	0.902	0.0
Nasopharyneal cancer	2	1.55(0.75,3.24)^c^	0.017	82.4	1.39(0.69,2.79)^c^	0.042	75.8	1.33(0.80,2.19)^c^	0.008	85.6
Melanoma	2	**0.79(0.64,0.97)**	0.765	0.0	0.96(0.77,1.21)	0.790	0.0	0.90(0.78,1.02)	0.725	0.0
Lung cancer	2	0.82(0.65,1.04)	0.852	0.0	1.07(0.82,1.40)	0.906	0.0	0.94(0.81,1.10)	0.984	0.0
Other cancers	7	1.08(0.96,1.22)	0.373	7.3	1.15(0.99,1.32)	0.747	0.0	1.08(1.00,1.17)	0.528	0.0
Ethnicity										
Asian	25	0.97(0.88,1.06)^c^	0.004	48.3	1.01(0.89, 1.15)^c^	0.003	49.3	0.99(0.93,1.05)^c^	0.030	37.8
Caucasian	13	1.03(0.95, 1.12)	0.120	32.8	1.00(0.92, 1.09)	0.277	16.5	1.01(0.96,1.06)	0.277	16.6
African	3	**1.72(1.24,2.38)**	0.288	19.6	1.23(0.78,1.95)^c^	0.039	69.1	1.25(0.90,1.74)^c^	0.022	73.9
Mixed	3	1.10(0.82, 1.48)	0.607	0.0	1.28(0.99, 1.65)	0.803	0.0	1.15(0.97,1.37)	0.610	0.0

a Number of comparisons.

b *P* value of *Q*-test for heterogeneity test.

c Random-effect model was applied when P value for heterogeneity < 0.05; otherwise, fixed-effect model was applied.

Statistically significant results were in bold.

**Figure 2:**
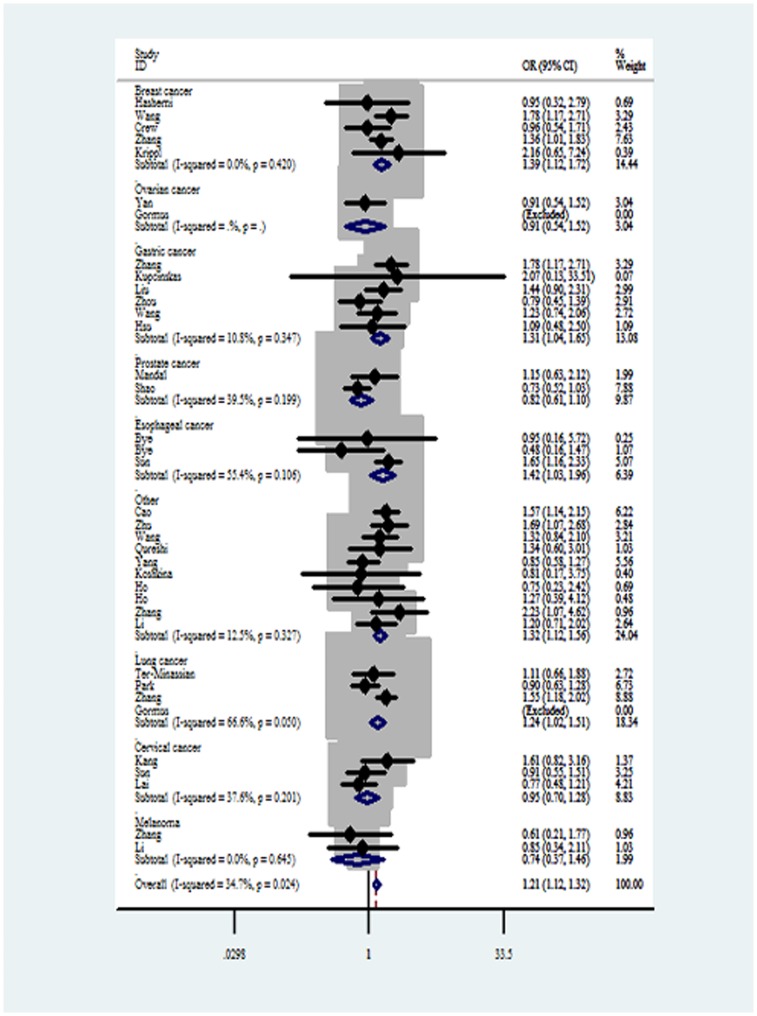
Forest plots of effect estimates for *Fas* -1377G>A polymorphism (AA vs GG). For each of the studies, the estimation of OR and its 95% CI is plotted with a *box* and a *horizontal line*. *Filled diamond* pooled OR and its 95% CI.

For *Fas* -1377G>A polymorphism, significantly increased cancer risks were observed in AA vs GG (Figure 2) and AA vs GA+GG comparison models in the overall analysis. In the subgroup analysis by cancer type, a significantly increased risk was observed in breast cancer for all comparison models. Meanwhile, increased risks were found for the comparison of AA vs GG and AA vs GA+GG in gastric cancer and esophageal cancer. In addition, a borderline decreased cancer risk was found in melanoma for GA vs GG and AA+GA vs GG comparison models (all data shown in Table 3).

**Table 3:** Stratified analyses of the *Fas* -1377G>A (rs2234767) polymorphism and cancer.

Variables	n^a^	AA vs GG			GA vs GG			AA+GA vs GG			AA vs GA+GG			A vs G		
		OR(95%CI)	*P* b	*I^2^*	OR(95%CI)	*P* b	*I^2^*	OR(95%CI)	*P* b	*I^2^*	OR(95%CI)	P^b^	*I^2^*	OR(95%CI)	P^b^	*I^2^*
Total	37	**1.19(1.06, 1.34)** c	0.024	34.7	1.00(0.94,1.06)^c^	0.033	32.2	1.03(0.97,1.10)^c^	0.012	37.8	**1.21(1.09, 1.34)** c	0.048	30.3	**1.06(1.00,1.11)** c	0.002	44.8
Cancer type																
Gastric cancer	6	**1.31(1.05, 1.65)**	0.934	0.0	0.99(0.85,1.14)	0.810	0.0	1.04(0.91,1.20)	0.659	0.0	**1.32(1.07,1.64)**	0.328	13.6	1.09(0.99,1.21)	0.372	7.0
Breast cancer	5	**1.39(1.12,1.72)**	0.420	0.0	**1.15(1.02,1.30)**	0.246	26.3	**1.18(1.06,1.32)**	0.253	25.3	**1.28(1.05,1.56)**	0.236	27.8	**1.15(1.06,1.26)**	0.186	35.3
Lung cancer	4	1.18(0.82,1.70)^c^	0.050	66.6	0.97(0.87,1.08)	0.743	0.0	1.01(0.91,1.12)	0.687	0.0	1.23(0.86,1.74)^c^	0.044	68.0	1.06(0.97,1.14)	0.279	21.8
Esophageal cancer	3	**1.42(1.03,1.96)**	0.106	55.4	0.96(0.66,1.37)^c^	0.031	71.2	1.00(0.72,1.39)^c^	0.043	68.1	**1.58(1.16,2.13)**	0.089	58.7	1.05(0.76,1.45)^c^	0.022	73.8
Cervical cancer	3	0.95(0.70, 1.28)	0.201	37.6	0.85(0.70,1.04)	0.149	47.4	0.88(0.73,1.06)	0.165	44.6	1.08(0.81,1.42)	0.215	35.0	0.95(0.83,1.09)	0.195	38.8
Prostate cancer	2	0.82(0.61,1.10)	0.199	39.5	0.91(0.53,1.54)^c^	0.042	75.9	0.90(0.54,1.50)^c^	0.042	75.8	0.91(0.70,1.19)	0.698	0.0	0.88(0.77,1.01)	0.099	63.2
Ovarian cancer	2	0.91(0.54,1.52)	NA	NA	1.04(0.77,1.40)	0.286	12.1	1.02(0.77,1.36)	0.270	17.9	0.91(0.56,1.50)	NA	NA	1.00(0.80,1.24)	0.308	3.8
Melanoma	2	0.74(0.37,1.46)	0.645	0.0	**0.79(0.62,1.00)**	0.614	0.0	**0.78(0.62,0.98)**	0.748	0.0	0.77(0.39,1.52)	0.617	0.0	**0.80(0.65,0.98)**	0.916	0.0
Other cancers	10	**1.32(1.12,1.56)**	0.327	12.5	1.07(0.97,1.17)	0.437	0.0	**1.10(1.01,1.21)**	0.364	8.5	**1.28(1.10,1.49)**	0.351	10.0	**1.12(1.04,1.20)**	0.244	21.7

a Number of comparisons.

b *P* value of *Q*-test for heterogeneity test.

c Random-effect model was applied when P value for heterogeneity <0.05; otherwise, fixed-effect model was applied.

Statistically significant results were in bold.

**Figure 3:**
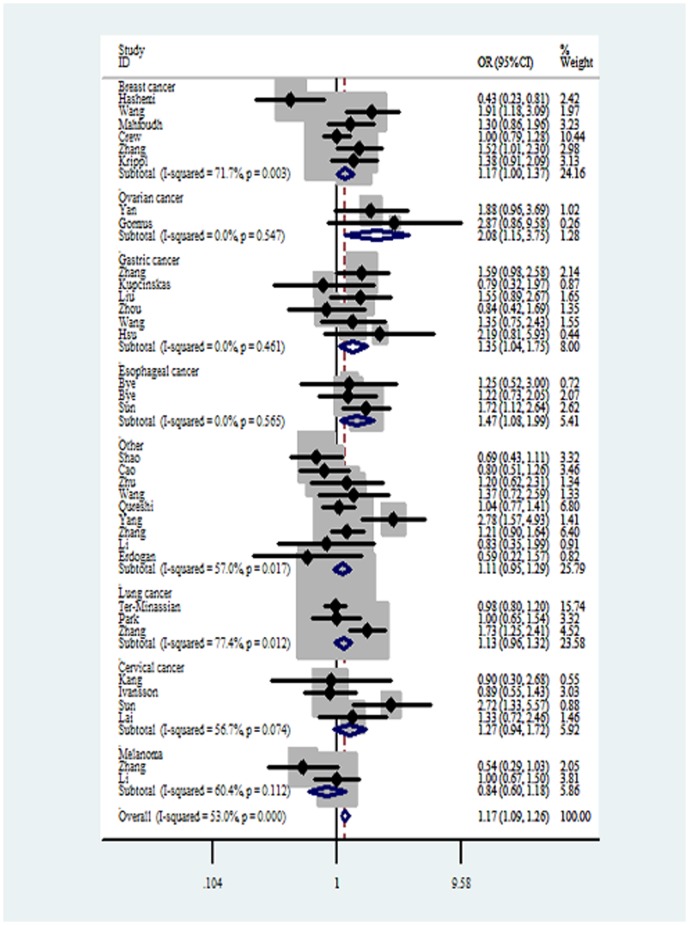
Forest plots of effect estimates for *FasL*-844C>T polymorphism (CC vs TT). For each of the studies, the estimate of OR and its 95% CI is plotted with a *box* and a *horizontal line*. *Filled diamond* pooled OR and its 95% CI.

For *FasL* -844C>T polymorphism, significantly increased cancer risks were observed in CC vs TT (Figure 3), CC+CT vs TT and CC vs CT+TT in the overall analysis. When the analysis was stratified by genotyping method, an increased cancer risk was observed in studies carried out by PCR-RFLP (shown in Table 4).

**Table 4:** Stratified analyses of the *FasL*-844C>T (rs763110) polymorphism and cancer.

Variables	n^a^	CC vs TT			CT vs TT			CC+CT vs TT			CC vs CT+TT			C vs T		
		OR(95%CI)	*P* b	*I^2^*	OR(95%CI)	*P* b	*I^2^*	OR(95%CI)	*P* b	*I^2^*	OR(95%CI)	*P* b	*I^2^*	OR(95%CI)	*P* b	*I^2^*
Total	35	**1.19(1.06, 1.35)** c	<0.0001	53.0	1.02(0.95,1.09)	0.135	21.2	**1.09(1.00,1.20)** c	0.046	30.6	**1.20(1.08, 1.34)** c	<0.0001	81.3	**1.13(1.05,1.22)** c	<0.0001	78.2
Genotype																
PCR-RFLP	24	**1.28(1.09,1.51)** c	0.001	53.4	0.97(0.88,1.08)	0.113	26.8	**1.14(1.03,1.25)**	0.063	32.7	**1.30(1.13,1.49)** c	<0.0001	83.3	**1.19(1.08,1.31)** c	<0.0001	79.6
TaqMan	8	1.04(0.92,1.18)	0.758	0.0	1.10(0.98,1.23)	0.842	0.0	1.07(0.97,1.19)	0.793	0.0	0.97(0.89,1.06)	0.839	0.0	1.01(0.95,1.07)	0.761	0.0

PCR-RFLP: restriction fragment length polymorphism.

a Number of comparisons.

b *P* value of *Q*-test for heterogeneity test.

c Random-effect model was applied when P value for heterogeneity <0.05; otherwise, fixed-effect model was applied.

Statistically significant results were in bold.

### Overall effects for alleles

Allele comparisons were also conducted in the meta-analysis. However, no significant associations were found in *Fas* -670A>G polymorphism and cancer risks (shown in Table 2).

There was borderline association between *Fas* -1377G>A polymorphism and cancer risks for A allele vs G allele in the overall analysis. In the subgroup analysis by cancer type, opposite results were shown between breast cancer and melanoma (shown in Table 3).

For *FasL* -844C>T polymorphism, in the subgroup analysis of genotyping method, an increased cancer risk was found in the studies carried out by PCR-RFLP (shown in Table 4).

### The *Fas* and *FasL* mRNA expression by genotypes and population

The *Fas* and *FasL* mRNA expression levels were stratified by genotype (shown in Table 5) and population (shown in Table 6) groups. In the genotype subgroup analysis, significant associations between mRNA expression levels and *Fas* -670A>G were observed in all populations (GA: *P* = 0.043), especially in Asian population (GG: *P* = 0.0003; dominant: *P* = 0.003; recessive: *P* = 0.001). Meanwhile, significant differences between mRNA expression levels and *FasL* -844C>T were observed in Asian population (recessive: *P* = 0.001). In the population-subgroup analysis, decreased expression of Fas was found in YRI (Yoruba in Ibadan) population than in the CEU population (*P* = 0.002).

**Table 5:** *Fas* and *FasL* mRNA expression by the genotypes of SNPs, using data from the HapMap^1^.

*Fas* -670A>G					*FasL* -844C>T				
Population	Genotypes	No.	Mean ± SD	*P* 2	Ethnicity	Genotypes	No.	Mean ± SD	*P* 2
CEU^3^	AA	23	8.79±0.36		CEU^3^	CC	76	5.94±0.07	
	GA	46	8.87±0.28	0.321		CT	5	5.89±0.07	0.137
	GG	12	8.74±0.36	0.687		TT	0	—	—
	Dominant	58	8.84±0.30	0.511		Dominant	5	5.89±0.07	0.137
	Recessive	69	8.84±0.31	0.292		Recessive	81	—	—
YRI^3^	AA	6	8.58±0.33		YRI^3^	CC	0	—	—
	GA	25	8.70±0.31	0.402		CT	28	5.94±0.06	—
	GG	53	8.67±0.30	0.450		TT	53	5.95±0.06	—
	Dominant	78	8.58±0.33	0.410		Dominant	81	5.95±0.06	—
	Recessive	31	8.67±0.31	0.987		Recessive	28	5.94±0.06	0.493
Asian^3^	AA	28	8.65±0.29		Asian^3^	CC	0	—	—
	GA	36	8.78±0.26	0.059		CT	50	5.96±0.06	—
	GG	21	8.98±0.30	**0.0003**		TT	33	5.91±0.06	—
	Dominant	57	8.85±0.29	**0.003**		Dominant	83	5.94±0.06	—
	Recessive	64	8.72±0.28	**0.001**		Recessive	50	5.96±0.06	**0.001**
All^3^	AA	57	8.70±0.33		All^3^	CC	76	5.94±0.07	
	GA	107	8.80±0.28	**0.043**		CT	83	5.95±0.06	0.163
	GG	86	8.76±0.33	0.297		TT	86	5.94±0.06	0.913
	Dominant	193	8.78±0.30	0.081		Dominant	169	5.95±0.06	0.390
	Recessive	164	8.76±0.30	0.871		Recessive	159	5.95±0.06	<0.0001

CEU: 90 Utah residents with ancestry from northern and western Europe; YRI: 90 Yoruba in Ibadan, Nigeria; Asian: 45 unrelated Han Chinese in Beijing and 45 unrelated Japanese in Tokyo.

1 Genotyping data and mRNA expression levels for *Fas* and *FasL* by genotypes were obtained from the HapMap phase II release 23 data from EBV-transformed lymphoblastoid cell lines from 270 individuals.

2 Two-side Student's *t* test within the stratum was used.

3 There were missing data for unavailable genotyping data.

Statistically significant results were in bold.

**Table 6:** *Fas* and *FasL* mRNA expression by the ethnicity, using data from the HapMap^1^.

*Fas* -670A>G				*FasL* -844C>T			
Ethnicity	No.	Mean ± SD	*P* 2	Ethnicity	No.	Mean ± SD	*P* 2
CEU^3^	81	8.83±0.31		CEU^3^	81	5.94±0.07	
YRI^3^	84	8.67±0.30	**0.002**	YRI^3^	81	5.95±0.06	0.120
Asian^3^	85	8.79±0.30	0.391	Asian^3^	83	5.94±0.06	0.398

CEU: 90 Utah residents with ancestry from northern and western Europe; YRI: 90 Yoruba in Ibadan, Nigeria; Asian: 45 unrelated Han Chinese in Beijing and 45 unrelated Japanese in Tokyo.

1 Genotyping data and mRNA expression levels for *Fas* and *FasL* by genotypes were obtained from the HapMap phase II release 23 data from EBV-transformed lymphoblastoid cell lines from 270 individuals.

2 Two-side Student's *t* test within the stratum was used.

3 There were missing data for unavailable genotyping data.

Statistically significant results were in bold.

### Test of heterogeneity

There was significant heterogeneity across the studies focused on these three polymorphisms as evaluated by Q-test. Then, we evaluated the heterogeneity for dominant model comparison by subgroups (cancer type, ethnicity, source of controls and genotyping method). As a result, ethnicity (*χ^2^* = 13.44, degree of freedom  = 3, *P_h_* = 0.004) and cancer type (*χ^2^* = 22.26, degree of freedom  = 11, *P_h_* = 0.022), but not source of controls (*χ^2^* = 1.49, degree of freedom  = 1, *P_h_* = 0.222) or genotyping method (*χ^2^* = 1.48, degree of freedom  = 4, *P_h_* = 0.830) contributed to substantial heterogeneity of the *Fas* -670A>G polymorphism. For the *Fas* -1377G>A polymorphism, the test revealed cancer type (*χ^2^* = 22.60, degree of freedom = 8, *P_h_* = 0.004), but not ethnicity (*χ^2^* = 4.81, degree of freedom = 3, *P_h_* = 0.187), source of controls (*χ^2^* = 0.42, degree of freedom = 1, *P_h_* = 0.518), or genotyping method (*χ^2^* = 0.51, degree of freedom = 3, *P_h_* = 0.917) contributed to substantial heterogeneity. For the *FasL* -844C>T polymorphism, genotyping method (*χ^2^* = 9.21, degree of freedom = 3, *P_h_* = 0.027), but not cancer type (*χ^2^* = 4.33, degree of freedom = 7, *P_h_* = 0.741), ethnicity (*χ^2^* = 5.64, degree of freedom  = 3, *P_h_* = 0.131), or source of controls (*χ^2^* = 0.08, degree of freedom  = 1, *P_h_* = 0.777) contributed to substantial heterogeneity.

### Sensitivity analyses

To assess the stability of the results and the source of the heterogeneity, sensitivity analysis was performed by sequential removal of each individual eligible study. For *Fas* -670A>G and *FasL* -844C>T polymorphisms, statistically similar results were observed after sequential removal of individual study in dominant and homozygote model, respectively, and the summary ORs in the other genetic models were not materially altered, suggesting that the results were stable. For the *Fas* -1377G>A polymorphism, sensitivity analysis indicated that study by Shao et al [38] was responsible for heterogeneity. The heterogeneity was decreased when this study was removed (AA+GA vs GG: *P_h_* = 0.075, *I^2^* = 26.5). Although the genotype distribution in 11 studies (listed in Table 1) didn't follow HWE, the corresponding summary ORs were not materially altered with or without including these studies for the three polymorphisms. In addition, no other single study altered the pooled ORs by sensitivity analysis.

### Publication bias

**Figure 4:**
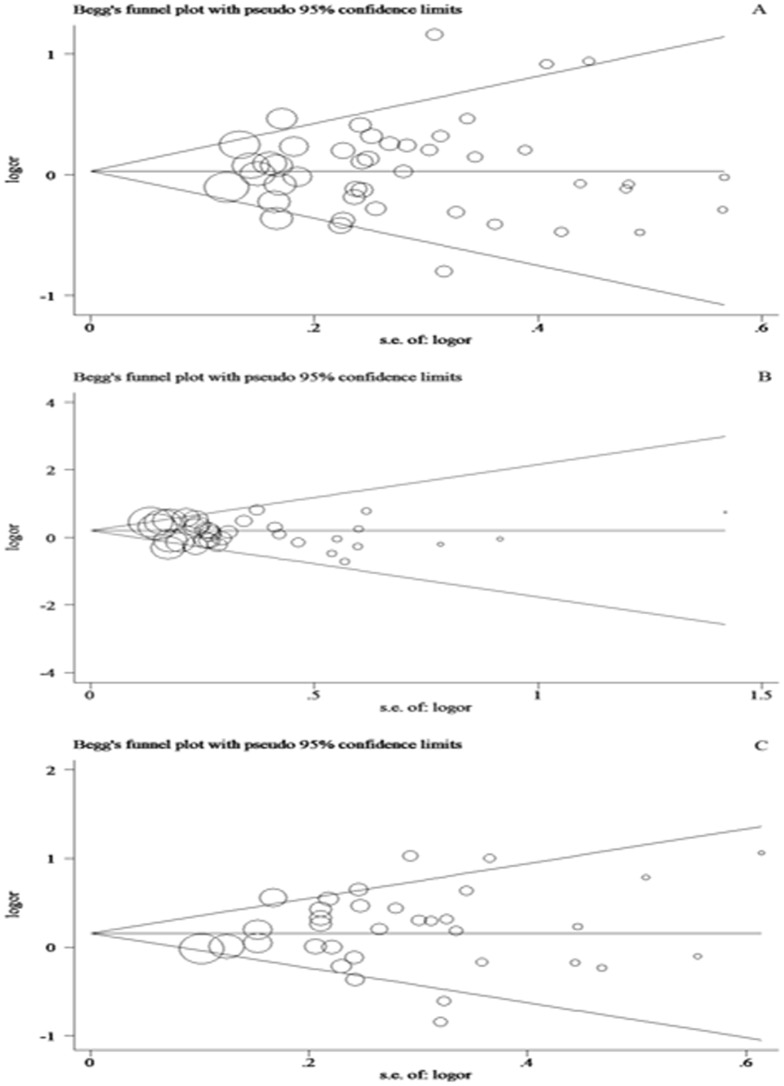
Begg's funnel plot of Egger's test for publication bias for three polymorphisms. Each circle represents as an independent study for the indicated association. Log[OR], natural logarithm of OR. Horizontal lines mean effect size. A: Begg's funnel plot of publication bias test for *Fas* -670A>G polymorphism. B: Begg's funnel plot of publication bias test for *Fas* -1377G>A polymorphism. C: Begg's funnel plot of publication bias test for *FasL* -844C>T polymorphism.

To assess the publication bias, Begg's funnel plot and Egger's test were performed and the shapes of funnel plots didn't show any obvious asymmetry in all genetic models of the three polymorphisms (Figure 4A–C). Therefore, to provide statistical evidence of funnel plot symmetry, Egger's test was performed for each of these polymorphisms and the results confirmed the absence of publication bias (*P*>0.05).

## Discussion

Fas, a potent member of the death receptor family, plays a crucial role in apoptotic signaling in many cell types [40]. Meanwhile, interactions between Fas and its receptor FasL trigger the death signal cascade, and subsequently induce apoptotic cell death [41]. Previous studies have indicated that down-regulation of Fas expression and/or up-regulation of FasL expression could be detected in many types of human tumors [42], [43]. The reason may be that down-regulation of Fas could protect tumor cells from elimination by anti-tumor immune responses, whereas up-regulation of FasL could increase the ability of tumor cells to counterattack the immune system by inducing apoptosis [44], [45], [46]. Therefore, it is believed that *Fas* and *FasL* play a crucial role in carcinogenesis. Given the important roles of *Fas* and *FasL* in carcinogenesis process, it is biologically plausible that *Fas* and *FasL* polymorphisms that possess the potential to influence the expression of Fas and/or FasL may be associated with cancer risk. Therefore, associations between the *Fas* -670A>G, *Fas* -1377G>A and *FasL* -844C>T polymorphisms and cancer risk were determined in this meta-analysis.

In this meta-analysis, 52 published studies were enrolled to determine the association between the three potentially functional polymorphisms within the *Fas* and *FasL* and cancer risk. This study revealed that the *Fas* -1377G>A and *FasL* -844C>T, but not the *Fas* -670A>G polymorphisms were associated with significantly increased overall cancer risk. Previous studies have identified that the -1377A allele had markedly reduced ability to bind transcription factor stimulatory protein 1 as compared with the -1377G allele, whereas the -670A and G alleles had similar ability to bind transcription factor signal transducers and activators of transcription 1 (STAT1)[47]. As the *Fas* -1377A allele reduced the ability to bind transcription factor stimulatory protein 1 that is a crucial transcriptional activator, the expression of Fas was decreased in carriers of the *Fas* -1377AA genotype as expected, but the *Fas* -670G allele didn't influence the expression of Fas [47], [48]. Therefore, it is reasonable that the *Fas* -1377A allele increased the overall cancer risk, and that the *Fas* -670G allele had no marked effect on overall cancer risk, which was consistent with our results. For the *FasL* -844T>C polymorphism, which is located in a binding motif for transcription factor CAAT/enhancer binding protein β, could influence the promoter activity of the *FasL* gene [49]. Additionally, it has been proposed that compared with the -844T allele, -844C allele strongly increased the expression of FasL on T cells and was associated with an enhanced rate of activation-induced cell death of T cells, which may lead to less powerful immune surveillance and increase the susceptibility to cancer [6].

The *Fas* -670GG genotype was associated with decreased risk of prostate cancer and melanoma according to the cancer type subgroup analysis. It was suggested that *Fas* -670A>G polymorphism might have the same effect on these two cancers. However, these results were based on 44 studies, which could affect the results owing to small amount of studies. Therefore, to draw a more precise conclusion, more related studies are needed.

For the *Fas* -1377G>A polymorphism, this study revealed that those who carried the -1377AA genotype had an increased risk for breast cancer, gastric cancer and esophageal cancer, while the melanoma risk was decreased. As described above, the different risk factors could contribute to the discrepancies. Also other unidentified causal genes would influence the effect of this polymorphism on different cancers.

For the *FasL* -844C>T polymorphism, the -844CC associated with increased cancer risk was observed in gastric cancer, esophageal cancer, and ovarian cancer among the previous studies, indicating that this polymorphism had similar effect on these three cancers. Although these cancers had different mechanisms of carcinogenesis, small amount of studies, publication bias, and other unidentified causal genes would be the result of the discrepancies, which contributed to the similar association between the *FasL* -844C>T polymorphism and three cancers.

In the subgroup analysis by ethnicity, an increased cancer risk in carriers of the *Fas* -670GG genotype was found in African, while the result of mRNA expression showed that GG genotype expressed higher levels of Fas in Asian populations. Meanwhile, the previous studies showed increased cancer risk in carriers of the *Fas* -1377AA and *FasL* -844CC genotype were found in Asian subjects, which was evidenced in mRNA expression by genotypes in Asian populations. However, this association was not proved in other ethnicities. The discrepancies in racial backgrounds and environment they lived in would lead to the differences. In addition, these polymorphisms might be masked by the presence of other unidentified causal genes involved in carcinogenesis. Due to the small size of population for the ethnicities, well-designed, large randomized case-control studies should be performed.

The pooled results of this study may be affected by polymorphism genotyping methods applied in the enrolled studies. Previous studies revealed that the pooled results of the *Fas* -670A>G polymorphism were not affected by the studies with genotyping methods of both PCR-RFLP and TaqMan. While *Fas* -1377AA genotype carriers increased cancer risk in the studies using PCR-RFLP but not TaqMan, and similar result was found in the *FasL* -844CC genotype carrier. The discrepancy across the studies applied different polymorphism genotyping methods may result from the different sensitivity and accuracy of genotyping methods. Meanwhile, the quality control is crucial to cause discrepancy as well. In general, studies [12], [17], [50] selected 10% repeated, random sample of subjects to test twice by standard genotyping method or different investigator, which was used to confirm the accuracy of results, while Mandal et al [51] and Ter-Minassian et al [22] tested 5% repeated samples. As a result, the consistency rate of quality control was 100% in almost all studies. However, the study by Crew et al [19] showed that the consistency rate was 100% for *Fas* -1377G>A, 94% for *Fas* -670G>A and 96% for *FasL* -844C>T. Therefore, the results of further studies should be confirmed by a standardized genotyping method. In addition, the limited amount of studies would also contribute to the discrepancy.

Heterogeneity is an important factor which can interpret the results of the meta-analysis. Therefore, we stratified the studies by cancer type, ethnicity, source of controls and genotyping method, respectively. The results showed that the main heterogeneity existed for cancer type and ethnicity. The reason might be that different cancers have different mechanisms of carcinogenesis. Virus infections, hormone levels, smoking, drinking, family history all could contribute to the different cancers. Meanwhile, different genetic backgrounds and different environmental factors among different ethnicities were the main factor of heterogeneity as well. Geographic differences, exposure of the Sun, eating habits, and environmental pollutes could exist in different ethnicities, which contributed to the heterogeneity.

Some limitations of the meta-analysis should be addressed. First, only studies in English were enrolled in this meta-analysis, which might miss some studies in other languages consistent with inclusion criteria. Second, some eligible studies included in the meta-analysis were hospital-based controls, which could generate the selection bias. Third, only a limited amount of studies was included, which might limit the strength of the associations. Finally, some suspected factors such as drinking, smoking, age, sex, and living habits were not considered in the meta-analysis. Regardless of such limitations, this meta-analysis still had some strengths. We investigated heterogeneity that may result from ethnicity of subjects, the types of cancer, the source of control subjects, and various genotyping methods. In addition, we analyzed the relationship between the mRNA expressions and genotypes, which partly supported the results of this meta-analysis.

In summary, this meta-analysis indicates that the *Fas*-1377G>A and *FasL* -844T/C polymorphisms are associated with increased cancer risk, but that no significant association is observed for the *Fas* -670A>G polymorphism and cancer risk. A definite conclusion should be made in the future through well-designed, unbiased, powered, population-based case–control association studies.

## Supporting Information

Checklist S1 **PRISMA Checklist.**
(DOC)
